# A New “Inhaler” for Sulphuric Ether

**Published:** 1866-05

**Authors:** 


*A New "Inhaler" for Sulphuric Ether.*—We have received a description of this instrument from its inventor, F. D. Lente, M. D., of Cold Spring, New York, and we are glad to learn, in this way, that our New York brethren are at last turning their attention towards sulphuric ether as a preferable anæsthetic to chloroform. In this instrument the ether is applied on a cone of flannel fitted upon a frame of light wire, and so adjusted as to be kept supplied with ether as occasion may require, without the necessity of removing it from the patient's face during the inhalation. Tieman & Co., of New York, are the manufacturers of the instrument.—*Boston Medical and Surgical Journal.*

